# Supplementary material for: Concordance among Swedish, German, Danish, and UK EQ-5D-3L Value Sets: Analyses of Patient-Reported Outcomes in the Swedish Hip Arthroplasty Register
Source: J Clin Med. 2021 Sep 17;10(18):4205. doi: 10.3390/jcm10184205 (PMC8471757; doi:10.3390/jcm10184205)
Supplement: Supplementary file 1 [file jcm-10-04205-s001.zip › jcm-1259663-supplementary.pdf]

Concordance among Swedish, German, Danish, and UK EQ-5D-3L value sets: analyses of patient-reported outcomes in the Swedish Hip Arthroplasty Register

Fitsum Sebsibe Teni[1*], Ola Rolfson[1,2,3], Jenny Berg[1], Reiner Leidl[4,5], Kristina Burström[1,6]

[1]Health Outcomes and Economic Evaluation Research Group, Stockholm Centre for Healthcare Ethics, Department of Learning Informatics, Management and Ethics (LIME), Karolinska Institutet, Stockholm, Sweden

[2]Department of Orthopaedics, Institute of Clinical Sciences, Sahlgrenska Academy, University of Gothenburg, Gothenburg, Sweden

[3]Swedish Hip Arthroplasty Register, Gothenburg, Sweden

[4]Institute for Health Economics and Health Care Management, Helmholtz Zentrum München, German Research Center for Environmental Health, Neuherberg, Germany

[5]Munich Center of Health Sciences, Ludwig-Maximilians University, Munich, Germany

[6]Equity and Health Policy Research Group, Department of Public Health Sciences, Karolinska Institutet, Stockholm, Sweden

[*]Corresponding author

Health Outcomes and Economic Evaluation research group, Stockholm Centre for Healthcare Ethics, Department of Learning Informatics, Management and Ethics (LIME), Karolinska Institutet, Stockholm, Sweden

Email: fitsum.teni@ki.se

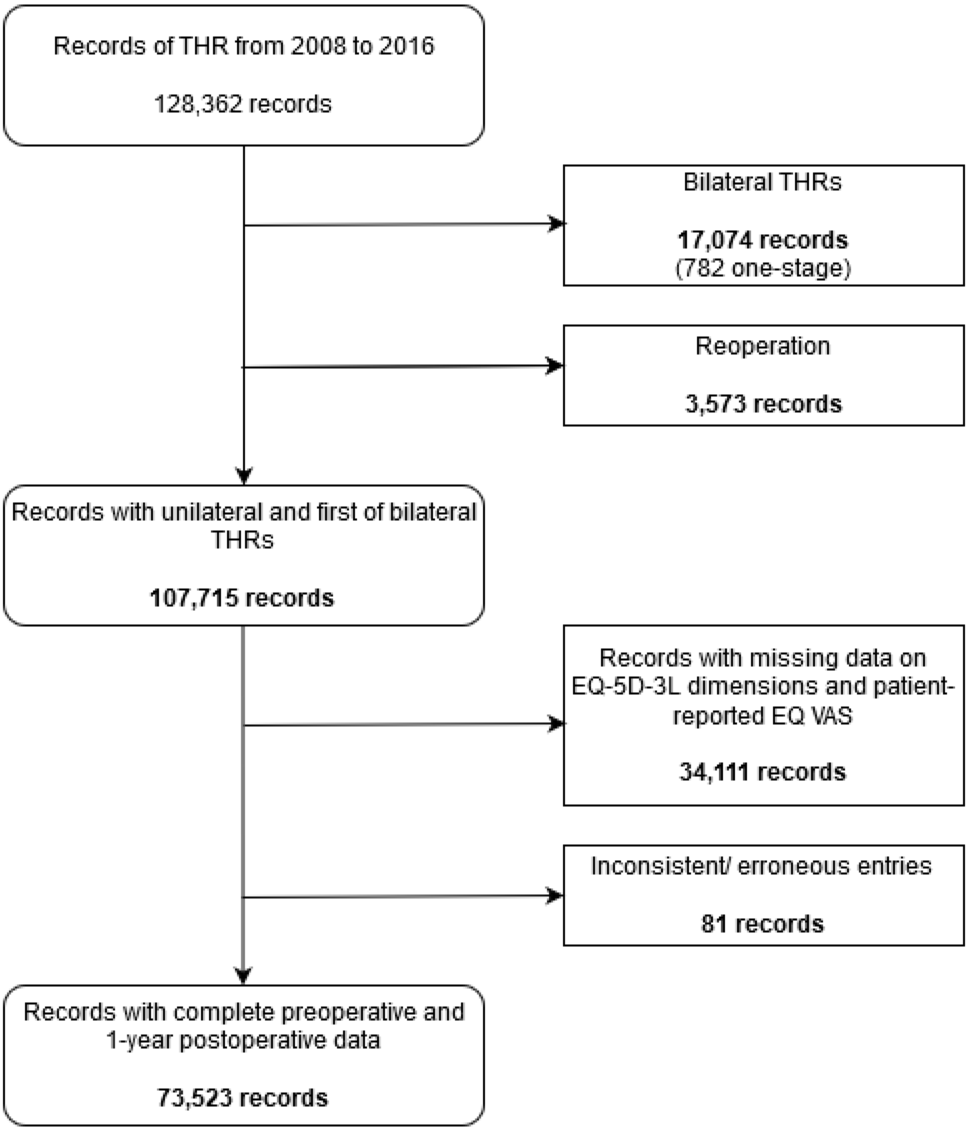

Figure S1. Data retrieval procedure

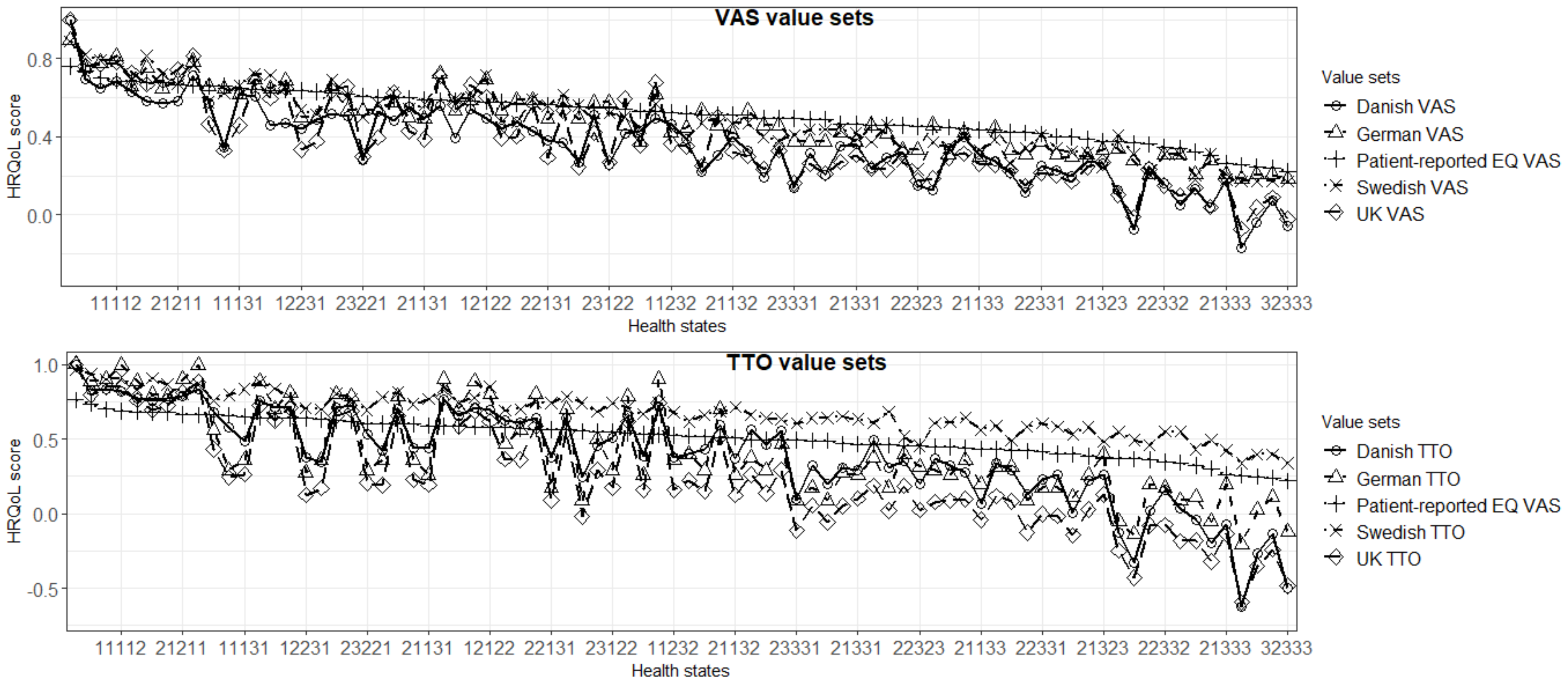

Figure S2. Comparison of patient-reported EQ VAS score with EQ-5D indices based on the value sets preoperatively (ordered by patient-reported EQ VAS) (n=73,523)

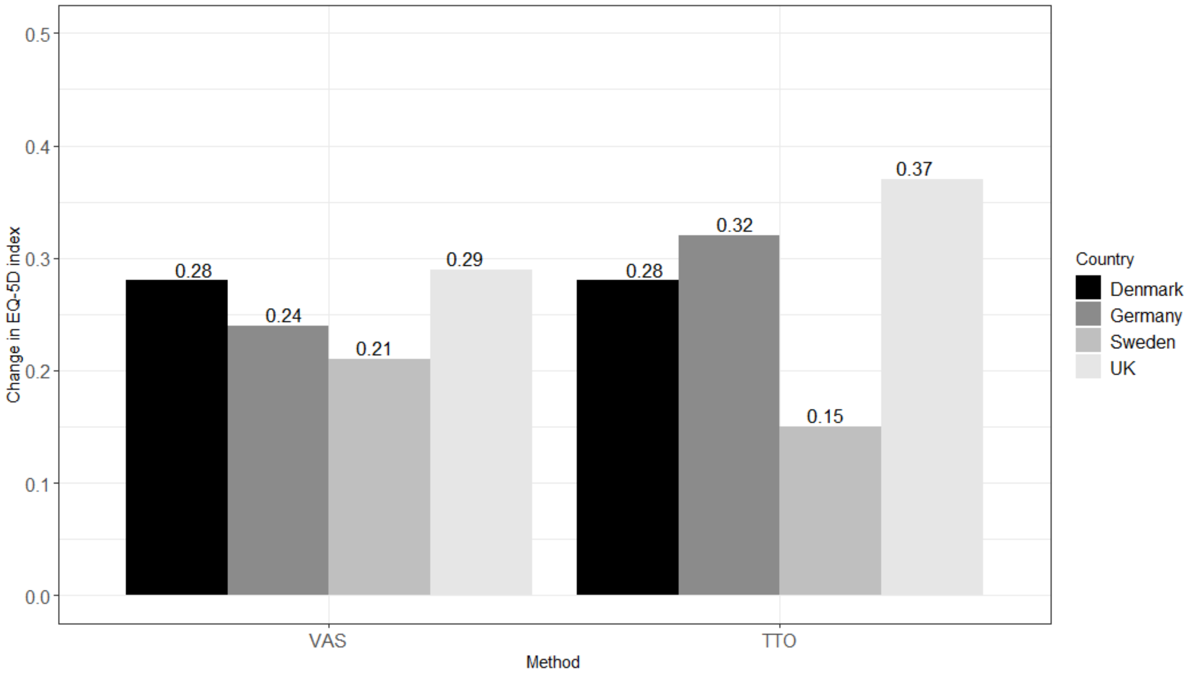

Figure S3. Mean change in EQ-5D index among value sets by method, 1-year postoperative follow-up

Table S1. Description of the value sets compared in the present study

Parameters	Value set							
	**Swedish VAS**[a]	**German VAS**[b]	**Danish VAS**	**UK VAS**	**Swedish TTO**	**German TTO**	**Danish TTO**	**UK TTO**
Perspective	Experience-based	Experience-based	Hypothetical	Hypothetical	Experience-based	Hypothetical	Hypothetical	Hypothetical
Number of health states	148	49 in 2006 and 58 in 2007	16 for each participant in 2 rounds of eight	45	148	36	46	42
Value for the health state 11111	0.887	0.893	1.000	1.000	0.969	1.000	1.000	1.000
Value for the health state 33333	0.172	0.184	-0.167	-0.073	0.340	-0.205	-0.624	-0.594
Sample size employed	Approximately 45,000	Nearly 2,000	1179	3,395	Approximately 45,000	339	1,332	3,395
Time of data collection	Surveys in 2004 and 2006	Surveys in 2006 and 2007	Winter 1999/2000	August to December 1993	Surveys in 2004 and 2006	October 1997 to March 1998	Spring 2000	August to December 1993
Model employed/ chosen	Ordinary least squares	Generalized linear model	Random effects model	Generalized least squares regression	Ordinary least squares	Additive linear model	Random effects model	Generalized least squares regression

[a]Scores divided by 100 to compare value sets [b]Final model was based on 2006 survey data

References: Burström et al, 2014; Leidl and Reitmeir, 2011; Szende et al, 2007; Greiner et al, 2005; Wittrup-Jensen, 2009; Dolan, 1997

Table S2. Pairwise comparisons of value sets undergone by method, perspective and country in the present study

Comparison	Value sets compared
Method	Swedish VAS vs. Swedish TTO<br>Danish VAS vs. Danish TTO<br>UK VAS vs. UK TTO
Perspective	Swedish VAS vs. Danish VAS<br>Swedish VAS vs. UK VAS<br>German VAS vs. Danish VAS<br>German VAS vs. UK VAS<br>Swedish TTO vs. German TTO<br>Swedish TTO vs. Danish TTO<br>Swedish TTO vs. UK TTO
Country	Swedish VAS vs. German VAS<br>Danish VAS vs. UK VAS<br>German TTO vs. Danish TTO<br>German TTO vs. UK TTO<br>Danish TTO vs. UK TTO

Table S3. Demographic and clinical characteristics of THR patients by sex preoperatively (n=73,523)

Variable	Sex					
	Total		Men		Women	
	%	n	%	n	%	n
**Age** (years)[mean(SD)]	68.2 (10.3)		67.0 (10.1)		69.2 (10.2)	
**Age group** (years)						
<50	4.6	3,408	5.8	1,839	3.8	1,569
50-59	13.4	9,852	15.6	4,957	11.7	4,895
60-69	34.3	25,215	36.1	11,458	32.9	13,757
70-79	34.6	25,443	32.2	10,219	36.4	15,224
80-98	13.1	9,605	10.3	3,281	15.1	6,324
**BMI category**[a]						
Underweight	0.7	489	0.2	56	1.0	433
Normal weight	29.6	21,783	23.8	7,572	34.0	14,211
Overweight	42.2	31,061	48.5	15,408	37.5	15,653
Obese group I	17.6	12,946	18.5	5,886	16.9	7,060
Obese group II	4.4	3,200	3.9	1,229	4.7	1,971
Obese group III	0.9	653	0.6	187	1.1	466
Missing	4.6	3,391	4.5	1,416	4.7	1,975
**ASA Class**						
Class I	25.0	18,402	26.5	8,423	23.9	9,979
Class II	57.8	42,517	55.0	17,464	60.0	25,053
Class III	13.7	10,050	14.9	4,727	12.7	5,323
Class IV	0.3	227	0.4	120	0.3	107
Missing	3.2	2,327	3.2	1,020	3.1	1,307
**Diagnosis**						
Primary OA[b]	92.5	68,022	92.9	29,508	92.2	38,514
Other[c]	7.4	5,473	7.0	2,235	7.8	3,238
Missing	0.0	28	0.0	11	0.0	17
**Type of surgery**						
Unilateral	83.5	61,389	84.9	26,961	82.4	34,428
Bilateral	16.5	12,134	15.1	4,793	17.6	7,341

a BMI categories: Underweight: <18.5; Normal: 18.5-24.9; Overweight: 25.0-29.9; Obese group I: 30.0-34.9; Obese group II: 35.0-39.9; Obese group III: > 40

b Osteoarthritis

c Other – Other secondary osteoarthritis, femoral head necrosis, sequele after childhood disease in the hip joint, inflammatory joint disease, other

Table S4. The forty most frequent health profiles recorded among THR patients pre- and 1 year postoperatively (n=73,523)

Preoperative				1-year postoperative			
**Health state**	**n**	**%**	**Cumulative percentage**	**Health state**	**n**	**%**	**Cumulative percentage**
21121	11,873	16.1	16.1	11111	27,843	37.9	37.9
21221	8,650	11.8	27.9	11121	10,516	14.3	52.2
21231	5,588	7.6	35.5	21121	8,734	11.9	64.1
21232	5,300	7.2	42.7	21221	4,040	5.5	69.5
21222	5,205	7.1	49.8	21222	3,477	4.7	74.3
21122	4,064	5.5	55.3	21122	2,866	3.9	78.2
21131	3,689	5.0	60.3	11122	1,934	2.6	80.8
22232	3,501	4.8	65.1	21111	1,733	2.4	83.2
11121	2,751	3.7	68.9	11112	1,331	1.8	85.0
22231	2,159	2.9	71.8	22222	1,227	1.7	86.6
22221	2,132	2.9	74.7	11221	1,044	1.4	88.1
22222	2,081	2.8	77.5	22221	799	1.1	89.1
21132	1,972	2.7	80.2	21232	790	1.1	90.2
22332	1,623	2.2	82.4	11222	500	0.7	90.9
21332	1,328	1.8	84.2	21231	436	0.6	91.5
21331	1,003	1.4	85.6	22232	423	0.6	92.1
22121	789	1.1	86.7	11211	347	0.5	92.5
22331	711	1.0	87.6	22121	347	0.5	93.0
11122	573	0.8	88.4	21211	333	0.5	93.5
11221	555	0.8	89.2	21131	279	0.4	93.8
21321	541	0.7	89.9	21112	263	0.4	94.2
11111	523	0.7	90.6	12111	215	0.3	94.5
21233	472	0.6	91.2	22122	214	0.3	94.8
21322	441	0.6	91.8	12121	212	0.3	95.1
22233	434	0.6	92.4	21132	212	0.3	95.4
22333	424	0.6	93.0	22322	159	0.2	95.6
22131	419	0.6	93.6	21223	158	0.2	95.8
22122	388	0.5	94.1	22332	153	0.2	96.0
11131	333	0.5	94.6	21322	144	0.2	96.2
22132	314	0.4	95.0	21332	140	0.2	96.4
22322	272	0.4	95.4	21212	134	0.2	96.6
21223	228	0.3	95.7	22233	126	0.2	96.7
22321	223	0.3	96.0	22231	117	0.2	96.9
21111	215	0.3	96.3	12221	116	0.2	97.1
11222	210	0.3	96.5	21321	111	0.2	97.2
21333	208	0.3	96.8	21233	105	0.1	97.4
21133	152	0.2	97.0	22223	104	0.1	97.5
21123	129	0.2	97.2	22333	82	0.1	97.6
11231	127	0.2	97.4	21123	76	0.1	97.7
22223	105	0.1	97.5	12222	73	0.1	97.8
**Sum**	**71,705**	**97.5**		**Sum**	**71,913**	**97.8**	

Table S5. Spearman's rank correlation and Lin's CCC tests of the full 243 health profiles between EQ-5D indices based on the value sets (n= 243)

Value sets compared	r_s [a]	95% CI [b]	Lin's CCC	95% CI
**Method**				
Swedish VAS vs. Swedish TTO	0.96	[0.96, 0.98]	0.55	[0.50, 0.60]
Danish VAS vs. Danish TTO	0.88	[0.84, 0.91]	0.76	[0.72, 0.79]
UK VAS vs. UK TTO	0.96	[0.95, 0.97]	0.70	[0.66, 0.73]
**Perspective**				
Swedish VAS vs. Danish VAS	0.76	[0.69, 0.81]	0.53	[0.46, 0.58]
Swedish VAS vs. UK VAS	0.76	[0.69, 0.81]	0.55	[0.48, 0.60]
German VAS vs. Danish VAS	0.73	[0.66, 0.78]	0.44	[0.38, 0.50]
German VAS vs. UK VAS	0.79	[0.74, 0.84]	0.46	[0.40, 0.52]
Swedish TTO vs. German TTO	0.62	[0.53, 0.70]	0.25	[0.21, 0.30]
Swedish TTO vs. Danish TTO	0.84	[0.80, 0.88]	0.29	[0.25, 0.33]
Swedish TTO vs. UK TTO	0.78	[0.71, 0.83]	0.18	[0.15, 0.21]
**Country**				
Swedish VAS vs. German VAS	0.86	[0.82, 0.89]	0.85	[0.81, 0.88]
Danish VAS vs. UK VAS	0.96	[0.95, 0.97]	0.92	[0.90, 0.94]
German TTO vs. Danish TTO	0.90	[0.86, 0.92]	0.88	[0.84, 0.90]
German TTO vs. UK TTO	0.95	[0.93, 0.96]	0.80	[0.76, 0.83]
Danish TTO vs. UK TTO	0.98	[0.96, 0.98]	0.86	[0.83, 0.88]

[a] Spearman's rank correlation coefficient
[b] Bootstrapped 95% CI
